# Improving Cutaneous Wound Healing in Diabetic Mice Using Naturally Derived Tissue-Engineered Biological Dressings Produced under Serum-Free Conditions

**DOI:** 10.1155/2024/3601101

**Published:** 2024-05-03

**Authors:** Meryem Safoine, Caroline Paquette, Gabrielle-Maude Gingras, Julie Fradette

**Affiliations:** Université Laval, Québec, QC, Canada

## Abstract

Long-term diabetes often leads to chronic wounds refractory to treatment. Cell-based therapies are actively investigated to enhance cutaneous healing. Various cell types are available to produce biological dressings, such as adipose-derived stem/stromal cells (ASCs), an attractive cell source considering their abundancy, accessibility, and therapeutic secretome. In this study, we produced human ASC-based dressings under a serum-free culture system using the self-assembly approach of tissue engineering. The dressings were applied every 4 days to full-thickness 8-mm splinted skin wounds created on the back of polygenic diabetic NONcNZO10/LtJ mice and streptozotocin-induced diabetic K14-H2B-GFP mice. Global wound closure kinetics evaluated macroscopically showed accelerated wound closure in both murine models, especially for NONcNZO10/LtJ; the treated group reaching 98.7% ± 2.3% global closure compared to 76.4% ± 11.8% for the untreated group on day 20 (*p*=0.0002). Histological analyses revealed that treated wounds exhibited healed skin of better quality with a well-differentiated epidermis and a more organized, homogeneous, and 1.6-fold thicker granulation tissue. Neovascularization, assessed by CD31 labeling, was 2.5-fold higher for the NONcNZO10/LtJ treated wounds. We thus describe the beneficial impact on wound healing of biologically active ASC-based dressings produced under an entirely serum-free production system facilitating clinical translation.

## 1. Introduction

Diabetes is one of the fastest-growing diseases of the century, affecting 537 million people worldwide in 2021, with a projected increase to 783 million in 2045, along with a rise of diabetes-related complications [1]. Diabetic foot ulcers are a chronic and debilitating condition affecting 40–60 million people globally [2], significantly reducing their quality of life and leading to lower limb amputation in 1% of diabetic patients [3]. Lower extremity complications represent an immense societal and economic burden, accounting for up to one-third of the 176 billion dollars yearly spent on direct medical expenses in the United States for diabetes care [4, 5].

The conventional treatment of diabetic foot ulcers integrates modalities, such as off-loading pressure from the ulcer along with blood glucose control, proper wound care consisting of regular debridement, appropriate inert dressing application, and infection treatment if required [6]. However, even with an adequate approach and the patient closely following off-loading recommendations, complete and lasting healing cannot be consistently achieved for all patients because the intrinsic characteristics of the skin wounds compromise the normal healing processes.

Many factors contribute to the perpetuation of chronic diabetic skin wounds, including sustained pro-inflammatory cytokine secretion [7] and impaired keratinocyte and fibroblast proliferation, migration, and differentiation [8, 9]. Other factors contributing to wound perpetuation include a disruption of the balance between extracellular matrix (ECM) accumulation and remodeling through matrix metalloproteinase dysregulated secretion and increased activity [10, 11]. A decrease in collagen I and III content [12] and overall impaired collagen secretion and assembly were also observed [13]. In addition, chronic diabetic skin is characterized by an impaired angiogenic response upon wounding (reviewed in [14]), mediated by altered secretion of growth factors such as basic fibroblast growth factor (bFGF), vascular endothelial growth factor (VEGF) and angiopoietin-1 (Ang-1) (reviewed in [15, 16]).

Several animal models have been described as mimicking many characteristics of the impaired wound healing observed in diabetic patients. One example is the chemical ablation of mouse pancreatic beta cells with streptozotocin (STZ) injections, which leads to the selective destruction of pancreatic beta cells and a rapid decline in insulin production, replicating the phenotype of type 1 diabetes [17–21]. Mice with STZ-induced diabetes display delayed wound closure, delayed reepithelialization, decreased neovascularization, and increased levels of pro-inflammatory cytokines, namely TNF and IL-6 [22–26]. The human pathogenesis of type 2 diabetes may be portrayed more accurately using polygenic obese models such as the NONcNZO10/LtJ mouse strain. These mice carry multiple susceptibility genes and develop diabetes only when exposed to an 11% high-fat diet [27]. These animals have been shown to display a delayed wound closure rate compared to nondiabetic controls [28].

There is an urgent need to improve the management of complex wounds in order to achieve permanent healing. In recent years, new treatments using mesenchymal stem/stromal cells (MSCs) have gained popularity, particularly using adipose-derived stem/stromal cells (ASCs), an easily accessible and abundant source of MSCs. Several studies have shown, in a preclinical setting, the benefits of ASCs to improve wound healing parameters, namely wound closure rate, reepithelialization, and angiogenesis [29–31]. ASCs can be delivered to the wound bed through local injections, topically applied cellular suspensions, or embedded in a biocompatible scaffold to be used as a biological dressing. A recent review comparing different ASCs' delivery systems for wound healing found that ASCs seeded in scaffolds were associated with better results than ASCs alone [32]. We have previously published the impact of adipogenic-differentiated human ASC-based dressings produced using the self-assembly approach, a process during which ASCs are stimulated with ascorbic acid to secrete and assemble endogenous matrix components, without the use of an exogenous scaffold [33]. These dressings were shown to promote wound healing in normal mice through stimulation of reepithelialization, granulation tissue formation, and angiogenesis. There are still no commercially available ASC-based dressings, although an ASC-based dressing has shown promising early clinical results in promoting wound healing in foot ulcers of diabetic patients [34]. The approved biological dressings for the treatment of diabetic foot ulcers, such as Apligraf® and Dermagraft® (Organogenesis), incorporate other cellular types, such as dermal fibroblasts with or without keratinocytes [35, 36]. Moreover, these products contain animal derivatives, which should be avoided in clinical products, especially fetal bovine serum (FBS). Many challenges are associated with the use of FBS, among which are safety concerns regarding the presence of infectious agents [37], lot-to-lot variability [38], and animal welfare [39]. Our team recently described a production system entirely devoid of FBS, from human ASC extraction to engineering of ASC-derived dressings displaying superior characteristics compared to the FBS-based counterparts [40]. These dressings produced in FBS-free conditions showed sustained pro-angiogenic molecule secretion, increased tissue thickness allowing easier manipulability, and a 50% reduction in production time [40]. We thus hypothesize that repeated application of these dressings would enhance repair through accelerated wound closure kinetics, reepithelialization, granulation tissue formation, and neovascularization. To do so, we evaluated the impact of these entirely serum-free ASC-derived biological dressings on wound healing outcomes of excisional dorsal skin wounds using two diabetic murine models, namely the polygenic NONcNZO10/LtJ mice and the more common chemically induced STZ-diabetic mice.

## 2. Materials and Methods

### 2.1. Isolation and Culture of Human ASCs

ASCs were isolated from human subcutaneous adipose tissue of women undergoing lipoaspiration or lipectomy procedures following their written informed consent. ASCs were extracted and cultured as previously described [40] before being frozen at passage (P) 0 using a serum-free cryopreservation medium CryoStor® CS10 (Stemcell™ Technologies, Vancouver, BC, Canada). A total of three ASC populations were used for biological dressings production (Table 1). The cells were extracted under completely xenogen-free serum-free conditions using the commercially available PRIME-XV MSC Expansion xenogen-free serum-free medium (XSFM) (FUJIFILM Irvine Scientific, Santa Ana, CA, United States) and PRIME-XV human fibronectin coating (FUJIFILM Irvine Scientific) [40]. ASCs, extracted under xenogen-free serum-free conditions, were extensively characterized in [40]. Before reaching confluency, cells were dissociated with TrypLE™ Select (Gibco, Thermo Fisher Scientific, Waltham, MA, USA. The mean age of donors was 38.3 ± 1.5 years, and the mean body mass index (BMI) was 25.6 ± 1.9 kg/m^2^ (Table 1).

### 2.2. Production of Cell-Based Living Biological Dressings

**Figure 1:**
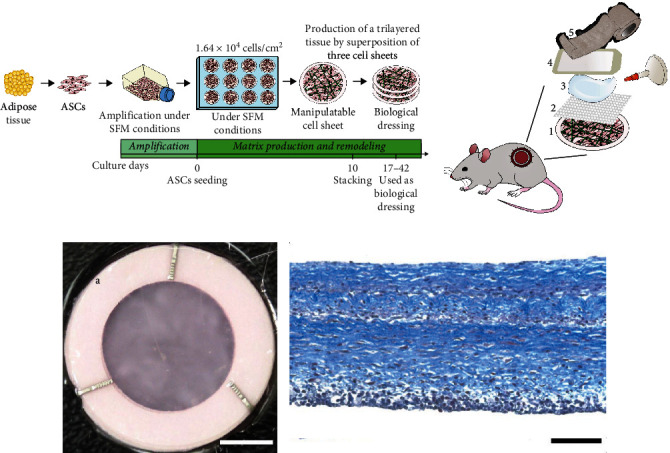
Schematic representation of the ASC-based biological dressings production method and *in vivo* experiments. (a) ASCs were extracted from human adipose tissues under serum-free conditions and cryopreserved, before being expanded in SFM and then seeded in 12-well plates. After 10–12 days of culture, manipulatable cell sheets were formed, lifted, and stacked in groups of three to form thicker tissues, which were applied as biological dressings on the surface of diabetic wounds. (b) Moist conditions were maintained during wound healing assessment using the following multilayer montage covering the wounds. Briefly, (1) a biological dressing was applied to the wounds of the treated group and omitted for the control animals. Then, all groups received in the following order, starting from inside-out, (2) a Mepitel® silicone mesh, (3) 0.1 ml of INTRASITE Gel, (4) a Tegaderm™ transparent film, and (5) a cohesive bandage. (c) Macroscopic appearance of an ASC-derived biological dressing (a = anchorage device), and (d) Histological aspect after Masson's trichrome staining of a biological dressing after 36 days of culture *in vitro* (24 days after stacking three cell sheets). Scale bars: (c) 0.5 cm and (d) 100 *μ*m.

Human ASC-derived biological dressings were produced using the self-assembly approach of tissue engineering as previously described in [40] and schematized in Figure 1(a), with the principal difference being a customized surface area to match wound size of approximately 1.2 cm^2^. ASC expansion and biological dressing production were conducted in a serum-free setting using the PRIME-XV MSC Expansion serum-free medium (SFM) (FUJIFILM Irvine Scientific). Proliferation data of the three ASC populations used for biological dressings production are presented in Table S1. Briefly, after thawing and amplification, ASCs were seeded at P2 or P3 in SFM medium with antibiotics (100 U/ml penicillin (Sigma–Aldrich, St. Louis, MO, USA), 25 *μ*g/ml gentamicin (GeminiBio, West Sacramento, CA, USA)) at a density of 1.64 × 10^4^ cell/cm^2^ in 12-well plates (Thermo Fisher Scientific) containing peripheric paper anchorage devices to prevent cell-mediated contraction *in vitro* (Whatman, Thermo Fisher Scientific). One day after cell seeding, the culture plates were positioned on a rotative platform (GyroTwister™, Thermo Fisher Scientific) to create a dynamic motion of the medium, with parameters set at a speed of 35 revolutions per minute (RPM) and a variation angle of 9° as previously reported in [41]. The culture medium was changed every 3 days. After 10–12 days of culture, the resulting cell and endogenously produced ECM sheets were stacked in groups of three to create trilayered biological dressings. These were kept in culture for a total of 17–42 days until being applied to the wounds of the animals. A subset of the unused biological dressings (*n* = 4) was processed on day 36 of culture for histological analysis. Briefly, the biological dressings were fixed in a 3.7% buffered formaldehyde solution, paraffin-embedded, and cross-sectioned (5 *μ*m) before being stained with Masson's trichrome. Images were taken using a Zeiss Axio Imager M2 microscope with an AxioCam ICc1 camera (Zeiss Canada, Toronto, ON, Canada). The dressing's conditioned media were also harvested for ELISA assays (Section 2.3).

### 2.3. Quantification of Angiomodulatory Molecules Secreted by ASC-Dressings Using ELISA Assays

Complete culture media conditioned by the ASC dressings were harvested after 20, 26, and 35 days of *in vitro* culture. Fresh media were conditioned for 48 hr before being frozen at −80°C until analysis. Controls consisted of incubating fresh medium for 48 hr in the absence of biological dressings. Conditioned media were analyzed for hepatocyte growth factor (HGF), plasminogen activator inhibitor-1 (PAI-1), VEGF, Ang-1, and bFGF secretion using Duoset® ELISA assays (R&D systems, Minneapolis, MN, USA) as per manufacturer instructions. Biological dressings produced using ASCs from three different donors were analyzed with three or four samples per donor per time-point. None of the listed molecules were detected in control samples at the assay threshold except for bFGF (mean level: 142 pg/ml), which was subtracted from the bFGF measured levels. Data are expressed as pg/mg of total protein content. The total protein content was determined in conditioned and control media using a BCA Protein Assay (Thermo Fisher Scientific).

### 2.4. Induction of Diabetes *In Vivo*

Animal experiments were performed at the animal facility of the research center of the CRCHU (QC, Canada). Two murine models were used for the wound healing experiments, namely the NONcNZO10/LtJ mice and the K14–H2B–GFP mice. The latter express green fluorescent protein (GFP) conjugated to histone 2B (H2B) under the keratin 14 (K14) promoter, displaying GFP-expressing keratinocytes, allowing to follow-up the reepithelialization process more accurately [42]. Ten NONcNZO10/LtJ mice were purchased from The Jackson Laboratory (The Jackson Laboratory, Bar Harbor, ME, USA) with a median age of 7 weeks and 5 days. Upon arrival at the animal facility, the mice were fed the Teklad S-2335 Mouse Breeder Sterilizable Diet (Envigo, Indianapolis, IN, USA), a high-fat diet containing 11.4% fat. Blood glucose levels were measured weekly using the OneTouch Ultra®2 blood glucose monitor (LifeScan, Milpitas, CA, USA) and the OneTouch Ultra® test strips (LifeScan). Blood glucose levels indicative of a diabetic state were reached after 13 weeks, with a diabetic threshold set at 14.0 mM.

Homozygous transgenic K14–H2B–GFP founder mice were initially provided by Dr. Elaine Fuchs (Rockefeller University, New York City, NY, USA), and a colony was established at the animal facility of the research center of CRCHU [33]. Diabetes was induced using a multiple low-dose STZ protocol as described in [43]. Male K14–H2B–GFP mice (total of 23 mice) between 8 and 10 weeks of age were included for the experiments. Briefly, mice were injected intraperitoneally with the equivalent of 50 mg/kg of STZ (Sigma–Aldrich) diluted immediately before the injection in a 50 mM sodium citrate buffer prepared using citric acid monohydrate ACS reagent grade (MP Biomedicals, Irvine, CA, USA) and sodium citrate dihydrate (Thermo Fisher Scientific). After the injection, the mice were provided 10% sucrose water prepared using sucrose (Bio basic, Markham, ON, Canada). The daily injections followed by 10% sucrose water were repeated for a total of 5 days, and on the sixth day, mice were returned to normal water. Blood glucose levels were measured the day before the first STZ injection and weekly afterward using the OneTouch Ultra®2 blood glucose monitor (LifeScan) and the OneTouch Ultra® test strips (LifeScan).

### 2.5. *In Vivo* Experiments

The wound healing experiments were performed when mice reached the hyperglycemic threshold, namely after 13 weeks of age for NONcNZO10/LtJ and 12–13 weeks for K14-H2B-GFP mice. NONcNZO10/LtJ mice were allocated in two equivalent groups (treated and untreated) of five mice for a total of 10 wounds per group. No attrition of the animal numbers occurred for the NONcNZO10/LtJ mice. For K14-H2B-GFP mice, two independent experiments were conducted, and data were combined for analyses. The first experiment (K14-H2B-GFP part A) included nine mice, four mice were allocated to the untreated group and five mice to the treated group. The second experiment (K14-H2B-GFP part B) included 11 mice, five mice were allocated to the untreated group and six mice to the treated group. ASCs populations used for dressing production are detailed in Table 1. Each mouse received biological dressings produced using ASCs derived from the same donor throughout the duration of the experiment. After combining data from the two K14-H2B-GFP experiments, two groups were formed, with untreated (*n* = 9) and treated (*n* = 11) animals, for a total of 18–22 wounds initially per group. Of note, out of the 11 K14-H2B-GFP mice of the treated group, one mouse died postsurgery on day 1. In contrast, for the untreated group (initially nine mice), six animals could not be followed until the final time-point. The reasons included postsurgical death (*n* = 1), excessive weight loss (*n* = 1), and removal/destruction of the anchoring silicone rings by the mice, which resulted in excessive contraction, thus preventing data comparison.

Animal manipulations were performed under isoflurane anesthesia. First, the skin of the back of the mice was shaved and depilated the day prior to the surgery. Two full-thickness wounds were formed by excising the skin, including the *panniculus carnosus* layer, on the mid-dorsum of each mouse using an 8 mm diameter biopsy punch (Acuderm Inc., Fort Lauderdale, FL, USA) as previously described [33, 44]. To prevent wound contraction, 0.5-mm thick silicone rings of 13 mm internal diameter cut from silicone sheets (Grace Bio-Labs, Bend, OR, USA) were centered and secured around the wounds using a medical grade immediate-bonding adhesive (Dermabond advanced®, Ethicon, Johnson and Johnson, New Brunswick, NJ, USA) and eight 5-0 polypropylene sutures (Prolene™, Ethicon, Johnson and Johnson) as inspired by Galiano et al. [45]. Immediately after surgery, a biological dressing was applied on each treated wound with the peripheral anchorage resting on the silicone ring. Both wounds on each mouse received the same treatment. The untreated group did not receive biological dressings but all the other components ensuring a moist environment (Figure 1(b)), namely from inside-out: (1) a 1 by 1 cm Mepitel® silicone mesh (Mölnlycke Health Care, Göteborg, Sweden), (2) 0.1 ml of INTRASITE Gel (Smith and Nephew, London, UK) and (3) a Tegaderm™ transparent film (3 M, Saint Paul, MN, USA) cut to fit each mouse. Finally, a cohesive bandage (Ritmed®, Medicom, Montreal, QC, Canada) was used to wrap and protect the wounds. The mice retained a full range of motion and were put in an individual cage. Every 4 days for NONcNZO10/LtJ mice and every 4 to 5 days for K14-H2B-GFP mice, coverage was removed under isoflurane anesthesia to take macroscopic pictures of the wounds (Section 2.6). At each time-point, after careful removal of the previous ASC dressings, new ASC-biological dressings were placed on the treated wounds, which were wrapped again as described in Figure 1(b).

### 2.6. Macroscopic Imaging of the Wounds and Global Wound Closure Analysis

On the day of surgery, at each dressing change, and at the end of the experiments, macroscopic pictures of each wound were taken with an EOS Rebel XSi camera (Canon Inc., Tokyo, Japan), including a ruler as a reference. The wound area was measured using the ImageJ software (National Institutes of Health). Global wound closure was expressed as the difference between the original wound area and the residual wound area at the specified time-points. The analyses were performed by two independent evaluators, with one of them being blinded to the experimental groups. The results were compiled using the mean of the measurements made by the two evaluators for each wound.

### 2.7. Fluorescent Imaging of the Wounds and Analysis

In addition to global macroscopic wound closure assessment every 4 to 5 days, the fluorescence emitted by the basal keratinocytes expressing GFP was also measured and served as an indicator of reepithelialization for the K14-H2B-GPF mice. The IVIS Lumina II In Vivo Imaging System (Caliper Life Sciences, A PerkinElmer Company, Hopkinton, MA, USA) was used for image acquisition on isoflurane-anesthetized mice with exposition times automatically set up by the system, and image analyses were performed using the device software. A scale of color was obtained ranging from yellow for strong signals to red for moderate signals to black for an absence of signal; the intensity of signals correlating with the presence of GFP-emitting keratinocytes. Reepithelialization rates (%) were calculated using the fluorescent reepithelialized area at specific time-points compared to the initial area of the wound devoid of fluorescence signal on the day of surgery.

### 2.8. Histological Analyses of Paraffin-Embedded Scar Tissues

On day 20 after surgery for NONcNZO10/LtJ mice, and days 20 and 22 for K14-H2B-GFP mice, the residual wounds/healed skin were harvested with surrounding margins of normal skin and fixed in 3.7% buffered formaldehyde solution and paraffin-embedded for histological processing. Cross sections (5 *μ*m) were stained with Masson's trichrome. Histological images were taken using the Zeiss Axio Imager M2 microscope with an AxioCam ICc1 camera (Zeiss Canada). Multiple images were acquired and stitched for each sample to allow the reconstitution of the entire tissue sample (complete wounds with adjacent margins) using the repositioning photomerge function of Photoshop CS4 software (Adobe, San Jose, CA, USA).

The thickness of the neoepidermis and the neodermis was measured for seven wounds from five animals per experimental condition for the NONcNZO10/LtJ mice on the Masson's trichrome stained tissues using the ImageJ software (National Institutes of Health). The respective skin margins of each wound were also measured. For the neoepidermis, wound areas on the histological section were analyzed in tiers; the two tiers on each side were referred to as the wound edges, and the central tier was referred to as central. The total neoepidermis was measured, including the *stratum corneum*. The thickness of the total epidermis of uninjured skin was also measured for the same NONcNZO10/LtJ mice on a portion of the skin far away from the wound margins (*n* = 10 mice). For the neodermis, the thickness measurements were made from the basal membrane (or the upper part of the wound if not completely closed) to the upper part of the loose connective tissue under the granulation tissue. For each sample, 20 measurements were made for the neoepidermis (10 for the central tier and five for each wound edge tier), and at least 10 measurements were made for the neodermis and the skin margins (five per side and combined). The mean thickness was then calculated for each sample.

Finally, for the NONcNZO10/LtJ mice, a validated histological wound healing score was calculated as described by Van de Vyver et al. [46] using six histological parameters, namely reepithelialization, epithelial thickness index, keratinization, granulation tissue thickness, remodeling, and the scar elevation index (*n* = 7 wounds per group). Each parameter was attributed either zero, one, or two points for a total maximum of 12 points, a score correlating with a completely closed and healed wound without excessive scarring [46].

### 2.9. Collagen Fiber Content Assessment Using Picrosirius Red Staining

Cross sections (5 *μ*m) of paraffin-embedded tissues were also processed for Picrosirius red staining (Direct Red 80, Sigma) to allow collagen content quantification. Images were taken using the Zeiss Axio Imager M2 microscope with an AxioCam ICc1 camera (Zeiss Canada) with a polarized light. Multiple images were taken per sample, and reconstitution of the entire sample was achieved using the Photoshop CS4 software (Adobe) and the repositioning photomerge function.

The histological collagen fiber content was quantified on the Picrosirius red-stained tissues from NONcNZO10/LtJ mice (*n* = 7 wounds per group) using the ImageJ software (National Institutes of Health) and the Color Inspector 3D plugin. The surface analyzed corresponded to the entire granulation tissue area. A graph was generated representing the color composition of the delimited area. A threshold was used to isolate the three main colors (red, yellow, green) seen in Picrosirius stained samples. Red represents mature collagen type I fibers, while green/yellow represents immature collagen type III fibers [47]. The relative proportion of each of these three colors was quantified by measuring the ratio of the pixel count of each color on the total pixel count for each image.

### 2.10. Immunolabeling on Tissue Cryosections

On the final day of the experiment, residual wounds/healed skin were also embedded in Tissue-Tek OCT compound (Sakura Finetek, Torrance, CA, USA), frozen in liquid nitrogen, and stored at −80°C. The samples were cut into 10 *μ*m transverse sections with a cryostat microtome (HM 550, Thermo Scientific, Ottawa, ON, Canada). Vascular density was assessed following CD31 immunostaining. Briefly, the samples (*n* = 7–9 wounds per group) were fixed in acetone and incubated with a rat primary antibody directed against endothelial cell marker CD31 (catalog number: 557355, BD Pharmingen, Mississauga, ON, Canada). The samples were then incubated with a donkey anti-rat secondary antibody conjugated to the Alexa 594 fluorophore (catalog number: A-21209, Life Technologies, Burlington, ON, Canada). A 5 mg/ml Hoechst solution (Sigma–Aldrich) was used for nuclei staining. For negative controls, the corresponding rat IgG2a antibody (catalog number: CBL605, EMD Millipore, Billerica, MA, USA) was used. Antibodies were diluted in phosphate-buffered saline containing 1% (mass/volume) bovine serum albumin (Sigma–Aldrich). The entire sample surface was acquired with an LSM 700 confocal microscope and the Zen 2010 software (Zeiss Canada) using the stitching mode. On these immunofluorescence images, a mean vascular area density was calculated using the ImageJ software, corresponding to the relative surface (%) occupied by CD31-positive structures within the entire granulation tissue area, from top to bottom. The color threshold range was set between 40 and 255. Seven to nine samples per group were analyzed using three cryosections per sample. The analyses were performed by two independent evaluators, with one of them being blinded to the experimental groups. The results were compiled using the mean of the measurements made by the two evaluators for each wound.

### 2.11. Statistical Analyses

Data are expressed as mean ± SD or mean ± SEM as indicated in the figure legends. Statistical comparisons were made using the GraphPad Prism 9 software (GraphPad Software Inc., La Jolla, CA, USA). The tests performed are specified in the figure legends (one-way analysis of variance (ANOVA) or unpaired *t*-tests). The confidence interval was set at 95% (*P* ≤ 0.05).

## 3. Results

### 3.1. Global Wound Closure of Diabetic Murine Skin is Enhanced by Human ASC-Based Biological Dressings

We assessed the therapeutic potential of biological living dressings engineered using a completely serum-free production system at each step of production, from ASC isolation and amplification to 3D reconstruction (Figure 1). This approach, based on the production and deposition of endogenous ECM from ASCs in culture, results in highly natural tissues featuring natural ECM and cells secreting bioactive molecules. The macroscopic appearance of ASC-derived biological dressings is shown in Figure 1(c). The engineered tissues are readily lifted from the culture plates and easy to manipulate using the peripheral anchorage device in order to subsequently be applied topically on wounds as trilayered dressings. On histological cross-sections, ASC-derived dressings show cells surrounded by an abundant ECM that is homogeneously distributed (Figure 1(d)).

The impact of repeated topical application of ASC-derived biological dressings on murine wound healing was evaluated using two diabetic mice models. Importantly, all mice reached hyperglycemic levels before the start of the experiments (Figure S1). On one hand, NONcNZO10/LtJ mice were subjected to a high-fat diet and their blood sugar levels were assessed weekly until reaching hyperglycemic levels established at 14.0 mM. After 13 weeks of a high-fat diet, mean blood sugar levels raised to 20.3 mM for the untreated group and 21.5 mM for the treated group (Figure S1(a)). Two mice had markedly elevated blood sugar above 30 mM compared to their NONcNZO10/LtJ counterparts, and one mouse was allocated to each of the treated or untreated group. On the other hand, diabetes was chemically induced using STZ for K14-H2B-GFP mice. After daily STZ injections, all K14-H2B-GFP mice became diabetic within 1 week, with mean blood sugar levels raising from 8.5 to 28.6 mM in the untreated group and from 8.3 to 28.3 mM in the treated group (Figure S1(b)). The K14-H2B-GFP mice in the treated or the untreated group had comparable blood sugar levels before induction of diabetes. After STZ injections, animals among both groups were equally diabetic with similarly high blood sugar levels (Figure S1(b)).

**Figure 2:**
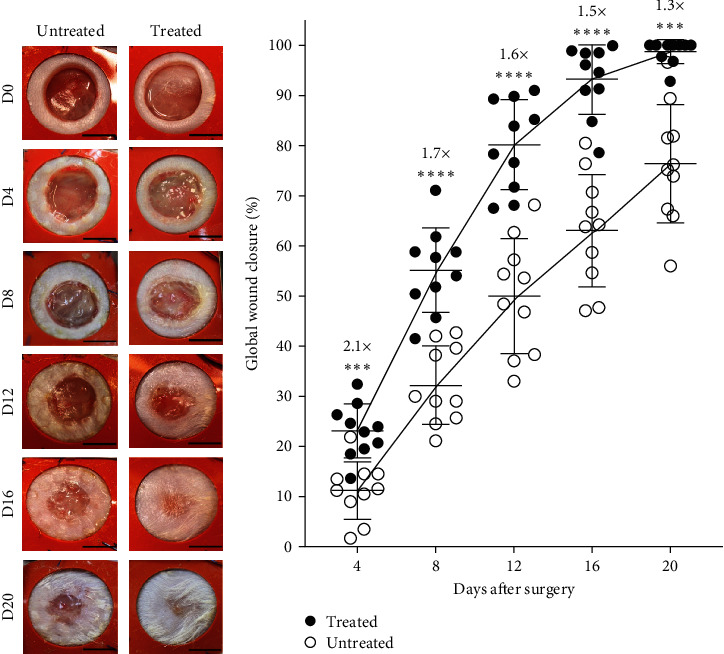
Global wound closure of NONcNZO/LtJ10 mice is accelerated by ASC-based biological dressings. (a) Macroscopic images of untreated (left column) and treated (right column) wounds on day (D) 0, D4, D8, D12, D16, and D20 after excisional surgery. Scale bars: 0.5 cm. (b) Kinetics of global wound closure percentage of untreated and treated wounds measured using macroscopic images, *n* = 10 wounds per group, mean ± SD, unpaired *t*-test with Welch's correction,  ^*∗∗∗*^ : *p*  < 0.001,  ^*∗∗∗∗*^ : *p*  < 0.0001.

After performing full-thickness skin excisional surgery on NONcNZO10/LtJ mice, global wound closure was evaluated macroscopically every 4 days over 20 days (Figure 2(a)). Wound closure was enhanced, especially at early time-points, with two distinguishable kinetics of healing for treated and untreated mice (Figure 2(b)). Indeed, global wound closure was enhanced as much as 2.1-fold in the treated group compared to the untreated one 4 days after surgery (Figure 2(b)). At the end of the experiment, namely on day 20, the treated group reached 98.7% ± 2.4% global wound closure while the untreated group only reached 76.4% ± 11.8% (*p*=0.0002). Since treated wounds exhibited a similar wound closure percentage (80.1% ± 9.0%) on day 12, this corresponds to an accelerated healing of at least 1 week for the wounds receiving the ASC-based dressings.

Global wound closure was also evaluated macroscopically for the STZ-induced K14-H2B-GFP mice, as presented in Figure S2(a). Global wound closure kinetics indicate an impact of the biological dressings as seen in the treated group at the earliest time-point 4 days after the surgery with a 1.9-fold percentage wound closure increase compared to the untreated group (Figure S2(b)). For other time-points, differences were not as striking, but statistical significance between groups was reached, namely on days 12–13 and at the end of the experiment, with a 1.2-fold and 1.1-fold increase, respectively, in wound closure. At the end of the experiment, the treated wounds exhibited a 94.6% wound closure compared to the untreated wounds at 84.5%. Of note, in the treated group, 10 K14-H2B-GFP mice out of 11 reached the final time-point while keeping the silicone rings intact (one post-operative death on day 1). However, in the untreated group, data from only three mice (six wounds) out of nine animals were included at the end of the study on days 20–22. Indeed, one death occurred on day 1, and one mouse had to be euthanized, considering excessive weight loss. In addition, other mice removed the silicone rings ensuring an adequate splinting of the wounds, thereby allowing wound contraction as the main mechanism of healing.

### 3.2. Treated Wounds Display a Complete and Well-Differentiated Epidermis along with Accelerated Early Reepithelialization

**Figure 3:**
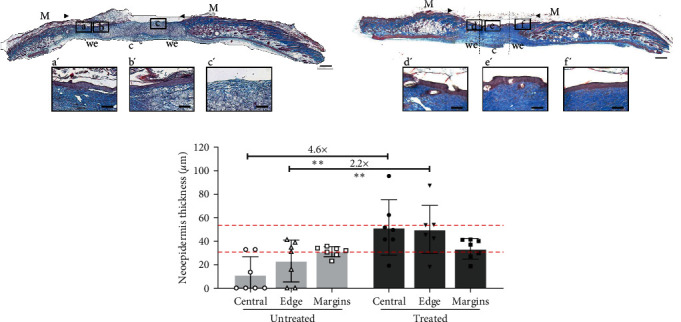
NONcNZO/LtJ10 mice treated wounds display complete reepithelialization. Representative examples of Masson's trichrome staining of wound/healed skin cross-sections 20 days after the excisional surgery for (a) untreated and (b) treated animals. Bars: 500 *µ*m. The wound margins (M) are indicated by the arrowheads. The wound edges (we) and the central (c) portion of the wounds are delimited by the dotted lines. (a′–f′) Magnifications of the areas delimited by the black boxes (a–f) are shown for each complete wound/healed skin section. Bars: 100 *µ*m. (c) Neoepidermis thickness was measured on histological cross-sections at the edges and central parts of untreated and treated wounds, as well as the epidermal thickness of wound margins. In comparison, the thickness of the epidermis of the uninjured skin from the same diabetic mice was measured and is presented as two dotted lines on the graph, representing the mean minimal and maximal thickness of the epidermis of uninjured skin (30.30 to 53.64 *µ*m, respectively). *n* = 7 wounds per group and *n* = 7 margins per group, mean ± SD, one-way ANOVA with Tukey post-hoc test.  ^*∗∗*^ : *p*  < 0.01.

**Figure 4:**
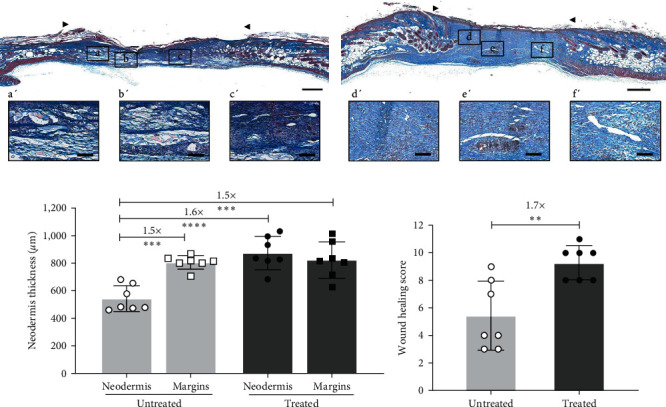
NONcNZO/LtJ10 mice treated wounds display thicker and homogenous granulation tissue along with improved overall healing features. Representative examples of Masson's trichrome staining of wound/healed skin cross-sections 20 days after excisional surgery for (a) untreated and (b) treated animals. Bars: 500 *µ*m. The wound area is delimited by the arrowheads. (a′–f′) Magnifications of the areas delimited by the black boxes (a–f) are shown for each complete wound/healed skin section. Bars: 100 *µ*m. (c) Neodermis thickness was measured on histological cross-sections of untreated and treated granulation tissues and of wound margins. *n* = 7 wounds per group and *n* = 7 margins per group, mean ± SD, one-way ANOVA with Tukey post-hoc test.  ^*∗∗∗*^ : *p*  < 0.001,  ^*∗∗∗∗*^ : *p*  < 0.0001. (d) The wound healing score was calculated by integrating six parameters from histological tissue sections. *n* = 7 wounds per group, mean ± SD, unpaired *t*-test with Welch's correction.  ^*∗∗*^ : *p*  < 0.01.

For histological analysis, wounds from NONcNZO10/LtJ mice were harvested along with adjacent skin (wound margins) after 20 days of healing of the excisional wounds, a time-point when the treated group reached an almost complete global wound closure of 98.7% ± 2.4% compared to 76.4% ± 11.8% for the untreated group. A large tissue section, including the full-thickness wound and its margins, was submitted for histological processing and Masson's trichrome staining in which ECM elements appear in blue and cellular components in pink. The histological comparison of wounds from untreated (Figures 3(a) and 4(a)) or treated (Figures 3(b) and 4(b)) NONcNZO10/LtJ mice shows distinctive features between the two groups with a better global wound appearance for the treated group in terms of reepithelialization and granulation tissue formation. For STZ-induced K14-H2B-GFP mice, tissues were harvested between days 20 and 22 of healing, and a similar histological comparison was made in Figure S3.

To analyze reepithelialization more precisely, the wound area was subdivided into three zones comprising the central wound region and two wound edge regions, which were also compared to the skin margins of each wound. The thickness of the neoepidermis was measured and compared between the different zones analyzed (Figure 3(c)).

Among the treated group for the NONcNZO10/LtJ mice, 100% of the wounds displayed complete reepithelialization. However, for the untreated group, only 43% of the wounds (three out of seven wounds) displayed complete reepithelialization, with the remaining wounds showing partial reepithelialization in the wound edge areas (two wounds) or no reepithelialization at all (two wounds). The central portion of the treated wounds displayed a 4.6-fold thicker epidermis compared to the untreated ones (Figure 3(c)) with a mean central thickness of 51.6 ± 23.6 *μ*m compared to 11.3 ± 15.5 *μ*m. Similarly, the wound edge areas indicated a 2.2-fold increase in thickness for the treated wounds (Figure 3(c)). More importantly, the thickness of the epidermis of the treated wounds did not differ from the epidermis of the adjacent skin margins (Figure 3(c)). As a comparison, the thickness of the uninjured skin from the same diabetic NONcNZO10/LtJ mice, further away from the wound margins, was also measured (mean of 42.43 ± 7.77 *µ*m, red dotted lines) and was similar to the thickness of the neoepidermis of the treated mice (Figure 3(c)).

Although histological results are not as contrasted as with NONcNZO10/LtJ mice, STZ-induced K14-H2B-GFP treated wounds displayed complete reepithelialization with a well-differentiated epidermis leading to the formation of a *stratum corneum*, along with homogenous granulation tissue (Figure S3(b)) compared to the untreated wounds (Figure S3(a)). The K14-H2B-GFP mice model allowed the specific assessment of reepithelialization using a noninvasive *in vivo* imaging of fluorescence since these mice feature a fluorescent epidermis and appendages resulting from nuclear GFP expression (GFP conjugated to histone 2 B) from the K14 promoter (Figure S4(a)). GFP expression is correlated with the signal intensity illustrated with a scale of colors with strong signals appearing in yellow and orange, moderate signals in bright red shades, and low to absent signals in dark red and gray (Figure S4(a)). The major benefit of biological dressings appears to be at the earliest time-point with a 1.9-fold increase in reepithelialization on day 4, the difference diminishing to 1.1- to 1.2-fold but remaining sustained at most subsequent time-points (Figure S4(b)). At the final time-point, the treated wounds displayed a slightly improved reepithelialization rate of 96.2% compared to 89.5% for the untreated wounds. These results need to be interpreted with caution because of the discrepancy between the number of mice in the two groups, as stated earlier.

### 3.3. NONcNZO10/LtJ Treated Wounds Exhibit a Homogenous Granulation Tissue Formation and Higher Collagen Content

Granulation tissue formation, another major phase of wound healing, was also analyzed using histological cross-sections of wounds from NONcNZO10/LtJ mice. The histological comparison of untreated (Figures 3(a) and 4(a)) and treated (Figures 3(b) and 4(b)) wounds show markedly denser and homogenous granulation tissue in the treated wounds. Granulation tissue thickness measurement revealed a 1.6-fold thicker granulation tissue for treated wounds compared to the untreated ones (Figure 4(c)). Interestingly, the neodermis of the treated wounds were comparable in thickness to the skin of the wound margins (Figure 4(c)).

**Figure 5:**
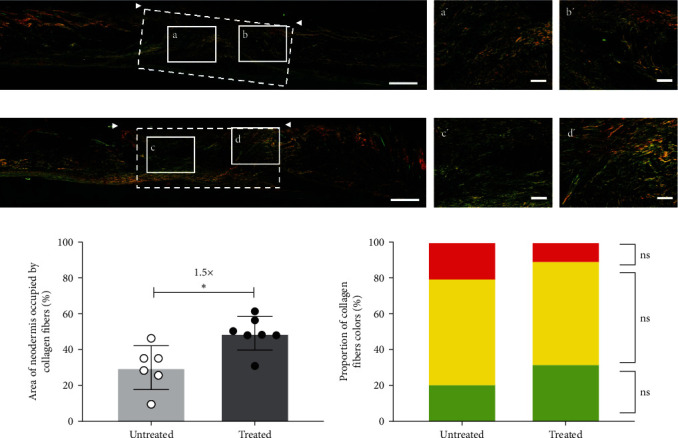
NONcNZO/LtJ10 mice treated wounds display higher collagen content with balanced collagen I/III proportions. Representative images of the (a) untreated and (b) dressing-treated wound/healed skin visualized after Picrosirius red staining of paraffin-embedded tissue sections on day 20 after surgery. Bars: 500 *µ*m. The wound area is indicated by the white arrowheads, and areas used for quantification are illustrated by the white dotted rectangles. (a′–d′) Magnifications of the areas delimited by the white boxes (a–d) shown in the complete tissue sections are presented on the right. Bars: 100 *µ*m. (c) percentage of the neodermis area occupied by collagen fibers. *n* = 6–7 wounds/group, mean ± SD, unpaired *t*-test with Welch's correction,  ^*∗*^ : *p*  < 0.05. (d) The proportion of green-, yellow-, and red-colored collagen fibers after subtraction of unoccupied space (black color). *n* = 6–7 wounds/group, mean ± SD, one-way ANOVA with Tukey's post-hoc test. No statistically significant differences were observed between the two groups for each color (green, yellow, red).

To further evaluate the granulation tissue quality of wounds from NONcNZO10/LtJ mice, collagen content and distribution were assessed using Picrosirius red staining (Figures 5(a) and 5(b)). Treated wounds featured a 1.5-fold higher total collagen content compared to the untreated ones (Figure 5(c)). The composition of the collagen content was also evaluated using the colors of the stained collagen fibers to distinguish between mature and immature fibers. Green/yellow fibers correspond to immature collagen, generally referring to collagen type III, and orange/red fibers correspond to mature collagen, generally referring to collagen type I. The analysis revealed rather similar proportions of green-, yellow-, and red-stained collagen fibers within treated and untreated wounds (Figure 5(d)). Both conditions displayed a balanced mixture of mature and immature fibers.

### 3.4. Treated Diabetic Wounds Exhibit an Improved Global Healing Score

To better integrate the impact of dressings on a maximum of wound processes, a comprehensive score was used to globally evaluate wound healing based on six histological parameters, namely reepithelialization, epidermal thickness, keratinization, granulation tissue thickness, scar elevation index, and remodeling, with a total scoring scale from 0 to 12. Treated wounds of NONcNZO/LtJ10 mice exhibited a higher wound healing mean score of 9.3/12 compared to the 5.4/12 score of untreated wounds (Figure 4(d)).

### 3.5. ASC-Derived Biological Dressings Promote Neovascularization

**Figure 6:**
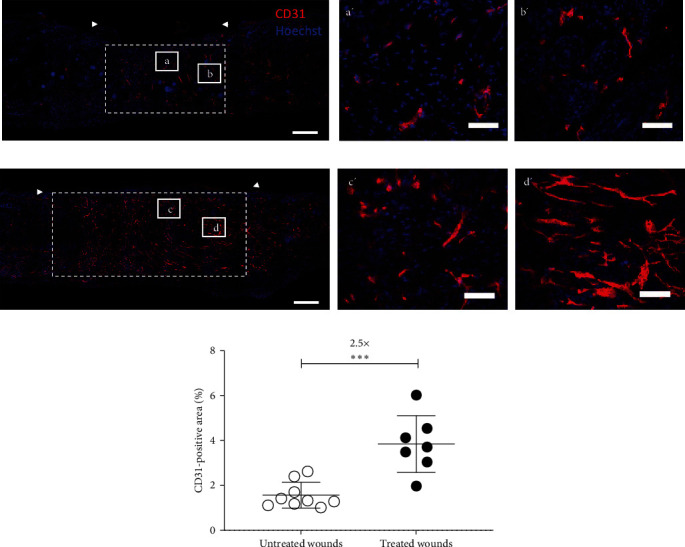
ASC-based biological dressings increase wound neovascularization in NONcNZO/LtJ10 mice. Confocal images showing CD31-labeled cells (red) and Hoechst nuclear staining (blue) staining of frozen wound cross-sections from (a) untreated and (b) dressing-treated animals 20 days after surgery. Bars: 500 *µ*m. The wound area is indicated by the white arrowheads, and areas used for quantification are illustrated by white dotted rectangles. (a'–d') magnifications of the areas delimited by the white boxes (a–d) shown in the complete tissue sections are presented on the right. Bars: 100 *µ*m. (c) Quantification of the percentage of the neodermis occupied by the CD31-positive structures. *n* = 7–9 wounds per group, mean ± SD, unpaired *t*-test with Welch's correction,  ^*∗∗∗*^ : *p*  < 0.001.

After 20 days of healing, neovascularization into the wounds of NONcNZO10/LtJ mice was evaluated using immunolabeling for CD31 on frozen tissue sections to identify capillary structures in the neodermis (Figures 6(a) and 6(b)). The area occupied by CD31-expressing structures within the neodermis was quantified, and a 2.5-fold increased vascular density was measured for the treated wounds compared to the untreated ones (Figure 6(c)). The global appearance of CD31-positive vascular structures also varied between treated and untreated conditions, with ASC-dressing treated wounds exhibiting long organized tubular structures (Figures 6(a) and 6(b)).

### 3.6. ASC-Derived Biological Dressings Display Stable Secretion of Bioactive Molecules

**Figure 7:**
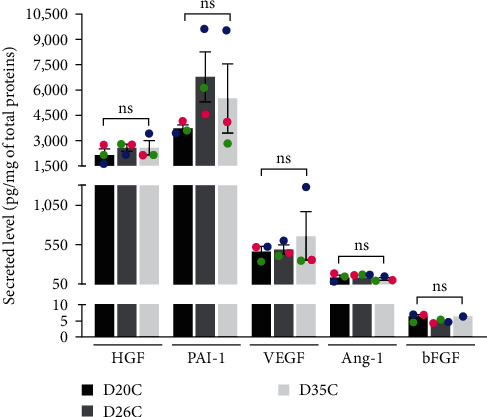
ASC-based biological dressings display a pro-angiogenic secretion profile. Quantification by ELISA of secreted levels of HGF, PAI-1, VEGF, Ang-1, and bFGF in media conditioned for 48 hr *in vitro* by the dressings. Each colored dot represents the mean values obtained for different donors. *N* = 3 ASC populations, *n* = 3–4 tissues per population per time-point. Of note, for bFGF at D35, the levels measured in the conditioned media were below the threshold control levels for two of the three ASC populations analyzed at the time-point. Mean ± SD, one-way ANOVA with Tukey's post-hoc test. No statistically significant differences were observed between the three time-points for all tested molecules.

To assess how the secretory activity of ASCs within the dressings impacted the wound healing processes, the secretory profile of five molecules involved in fibroblast recruitment and proliferation, as well as angiogenesis, were measured by ELISA (Figure 7). High levels of secreted HGF, PAI-1, VEGF, Ang-1, and bFGF were measured in conditioned media *in vitro*, for tissues engineered with ASCs from each of the three donors (Table 1). Furthermore, considering that all dressings used for the *in vivo* study were engineered at the same moment, but new dressings were applied every 4 days at the wound surface of mice during the healing period, the dressing's functionality was assessed over time. The secreted levels of each growth factor remained stable for dressings maintained in culture for 20, 26, or 35 days (Figure 7).

## 4. Discussion

Cell-containing biological dressings represent a promising strategy to promote the healing of diabetic wounds, providing a platform where matrix-embedded cells and secreted molecules are partly protected against the proteolytic environment of diabetic wounds [48]. In recent years, ASCs have emerged as great candidates to promote wound healing both at preclinical and clinical levels, with improved diabetic wound outcomes often associated with their wide range of secreted bioactive molecules [49].

Among the challenges associated with the use of biological products in a clinical setting is the preponderance of animal-derived products, namely FBS. Commercially available products such as Apligraf® and Dermagraft®, approved for the treatment of diabetic foot ulcers, are produced using FBS. However, safety concerns, lot-to-lot variability in quality and composition, limited availability, as well as animal welfare warrant the search for FBS alternatives and replacement of animal derivatives when considering clinical translation, as recently reviewed [50]. Our work describes the *in vivo* impact of dressings engineered using a method resulting in ASC-based tissues devoid of FBS from ASC extraction to tissue production [40]. In addition to a serum-free medium, a recombinant enzyme instead of porcine trypsin and a serum-free cryopreservation agent were also used [40]. FBS substitution without affecting ASCs' phenotypic and functional properties is challenging. The detailed characterization of ASCs extracted under serum-free conditions in Safoine et al. [40] showed maintained surface markers (CD44, CD73, CD90, and CD105), along with enhanced properties of ASC-derived tissues featuring increased thickness and mechanical properties.

In the present study, we assessed the effects of these ASC-based tissues, produced under a completely serum-free system, on the cutaneous repair of full-thickness splinted wounds of diabetic mice when applied as temporary dressings. We used two relevant murine models of diabetes: polygenic diabetic NONcNZO10/LtJ mice and chemically induced diabetic K14-H2B-GFP mice, representative of human type 2 and type 1 diabetes, respectively. Both models exhibit wound healing impairment, which is well documented [22, 26, 28, 51]. For example, Blaber et al. [28] showed that NONcNZO10/LtJ mice displayed a 2.7-fold reduced wound closure rate of splinted full-thickness wounds when compared to nondiabetic control mice. Interestingly, a comparative study performed by Fang et al. [51] showed that diabetes led to more pronounced skin healing impairments in the NONcNZO10 strain than STZ-induced mice compared to their respective non diabetic controls, especially for excisional wounds.

Our data show that ASC-based dressings applied regularly on splinted full-thickness wounds of diabetic mice allow faster wound closure compared with untreated wounds. This difference was observed for NONcNZO10/LtJ and K14-H2B-GFP mice, although being more pronounced for treated NONcNZO10/LtJ mice. The difference was noticeable as early as the first dressing change on day 4. Recent studies using repeated applications of different types of ASC-based formulations have shown accelerated wound closure [52, 53]. Indeed, Xia et al. [52] studied the effect of ASCs incorporated in a gelatin hydrogel on wounds of STZ-induced diabetic mice and found a macroscopic closure of 95% on day 21 compared to 75% for the hydrogel alone [52]. Consistent with these results, we have observed comparable closure rates on days 20–22 in both murine models; treated NONcNZO10/LtJ mice displaying 98.7% compared to 76.4% for the untreated mice and treated STZ-induced K14-H2B-GFP mice displaying 94.6% compared to 84.5% for their untreated counterparts. In the second study, Sotelo Leon et al. [53] reported the effect of ASCs seeded in a collagen hydrogel produced from decellularized cadaveric human tendons using a splinted full-thickness wound model in diabetic rats [53]. Their results show an impact on global wound closure at early time-points (51% vs. 39% on day 6, 62% vs. 51% on day 8, and 79% vs. 67% on day 12 for treated and untreated wounds, respectively), with no effects at the end of their study on day 16 [53].

Along with faster global wound closure, our study revealed the impact of ASC-based dressings on different processes of wound healing (reepithelialization, granulation tissue formation, and angiogenesis). The longitudinal assessment of reepithelialization is challenging on macroscopic and histological images. We used mice with GFP-expressing keratinocytes to specifically follow keratinocyte migration onto the wound in a noninvasive fashion through the imaging of the fluorescence emitted by the epidermis of STZ-induced K14-H2B-GFP mice. Despite the lower number of mice in the untreated group able to reach the final time-point (*n* = 6 wounds), data from this experiment is valuable in providing specific insight into reepithelialization. Our results reveal a favorable impact of ASC-based dressings on wound reepithelialization in these mice, with the largest effects observed in the early days of skin repair (days 4–9). This conclusion is also supported with data from the NONcNZO10/LtJ mice model: the histological analyses of the neoepidermis on day 20 revealed a complete reepithelialization for all treated wounds in comparison to less than half (43%) of the untreated wounds, along with increased epidermis thickness for the treated wounds. Our results are aligned with other reports showing the beneficial effects of ASC-based dressings on reepithelialization and epidermis thickness [52, 54–56].

The formation of granulation tissue is another process greatly promoted by ASC therapy, as reported in our study with an increase of the granulation tissue thickness along with a more homogenous and organized aspect among the treated wounds of the NONcNZO10/LtJ mice when compared to the untreated ones. Other groups noticed similar effects on granulation tissue formation [52, 54, 56, 57]. Jiang et al. [54] studied the impact of ASC-loaded porcine intestinal submucosa on wound healing of diabetic rats and found increased granulation tissue thickness on day 28 (1.4 vs. 1.0 mm) when compared to untreated wounds, with a similar collagen content between treated and untreated wounds when evaluated using Picrosirius Red staining [54]. Tyeb et al. [57] evaluated ASCs embedded in gelatin scaffold and described an increase in collagen deposition in treated wounds of STZ-induced diabetic rats along with a dramatic increase in the ratio of collagen I/III evaluated with Picrosirius Red staining when compared to untreated wounds [57]. In our study, an increase in collagen content in treated wounds was quantified without a noticeable change in the collagens ratio, with both treated and untreated wounds displaying a balanced collagen composition.

During the proliferative phase of wound healing, ASCs also promote angiogenesis within the wounds through the secretion of pro-angiogenic factors, especially VEGF, Ang-1, and HGF [29, 58, 59]. We have shown that ASC-based dressings increase the vascular density (2.5-fold) in the granulation tissue and that the new capillary network is more organized. Several studies have documented the beneficial effects of ASC-loaded scaffolds on neoangiogenesis of full-thickness wounds of various models of diabetic rodents; the vascular density of treated wounds increasing 1.4- to 6-fold when compared to wounds not receiving ASCs [53, 55, 56, 58, 60–63]. We assessed the secretion levels of multiple molecules involved in the stimulation of the wound healing proliferative phase processes with an emphasis on pro-angiogenic factors. Our ASC-based dressings maintained steady secretion levels of VEGF, Ang-1, HGF, PAI-1, and bFGF throughout the culture period corresponding to the time that the first and the last dressings were applied on mice.

In conclusion, our data indicate that all-natural human ASC-based self-assembled dressings acted not only on the speed but also on the quality of the wound healing process in these preclinical studies. Indeed, the detailed analysis of the healed skin of NONcNZO10/LtJ mice showed a well-differentiated epidermis, a thicker and more homogenous granulation tissue enriched in collagen and new blood capillaries in the treated wounds compared to the untreated ones. The complementary STZ-induced K14-H2B-GFP model provided insight into the beneficial effects of ASC dressings on reepithelialization, especially at early time-points. While our study does not allow to conclude on a potential differential response to the ASC dressings between the type 1 vs. type 2 diabetic murine models, the enhanced skin repair observed for treated wounds of the NONcNZO10/LtJ animals is particularly significant considering the relevance of this polygenic model of type 2 diabetes to recapitulate features of impaired skin healing [51]. The clinical relevance of this diabetic model is further supported by the higher prevalence of type 2 diabetic patients to develop chronic foot ulcers [64]. Of note, the entire dressing production was performed without the use of animal serum from extraction to tissue production using the self-assembly approach of tissue engineering, leading to natural tissues rich in human cells and matrix components. These new ASC-based dressings thus enhance diabetic wound healing at a preclinical level with the distinctive feature of being highly relevant for clinical translation.

## Figures and Tables

**Table 1:** Description of the characteristics of human donors from which cells were extracted for biological dressing production.

Population ID number	Source	Age of donor (years)	BMI of donor (kg/m^2^)	Corresponding color in Figure 7	Animal experiments (number of mice that received biological dressings)
ASCs#1	Lipoaspiration	40	26.3	Green	NONcNZO10/LtJ (*n* = 5) K14-H2B-GFP Part B (*n* = 3)
ASCs#2	Lipoaspiration	38	27.1	Pink	K14-H2B-GFP Part B (*n* = 3)
ASCs#3	Lipectomy	37	23.5	Blue	K14-H2B-GFP Part A (*n* = 5)
Mean (SD)	—	38.3 (1.5)	25.6 (1.9)	—	—

ASCs, adipose-derived stromal/stem cells; BMI, body mass index; SD, standard deviation.

## Data Availability

All data that support the findings of this study are included within the article and the supplementary file.

## Supplementary Materials

Supplementary Materials Table S1: population doubling levels of the ASCs used for biological dressing production. Figure S1: all NONcNZO/LtJ10 and K14-H2B-GFP mice reached diabetic blood glucose level threshold. Figure S2: ASC-derived biological dressings accelerate global wound closure notably in early phase in STZ-induced diabetic K14-H2B-GFP mice. Figure S3: wounds of STZ-induced diabetic K14-H2B-GFP mice treated with biological dressings display complete reepithelialization and homogenous granulation tissue. Figure S4: ASC-derived biological dressings accelerate early reepithelialization in STZ-induced diabetic K14-H2B-GFP mice.
