# Supplementary material for: Improving Cutaneous Wound Healing in Diabetic Mice Using Naturally Derived Tissue-Engineered Biological Dressings Produced under Serum-Free Conditions
Source: Stem Cells Int. 2024 May 3;2024:3601101. doi: 10.1155/2024/3601101 (PMC11087150; doi:10.1155/2024/3601101)
Supplement: Supplementary Materials — Table S1: population doubling levels of the ASCs used for biological dressing production. Figure S1: all NONcNZO/LtJ10 and K14-H2B-GFP mice reached diabetic blood glucose level threshold. Figure S2: ASC-derived biological dressings accelerate global wound closure notably in early phase in STZ-induced diabetic K14-H2B-GFP mice. Figure S3: wounds of STZ-induced diabetic K14-H2B-GFP mice treated with biological dressings display complete reepithelialization and homogenous granulation tissue. Figure S4: ASC-derived biological dressings accelerate early reepithelialization in STZ-induced diabetic K14-H2B-GFP mice. [file 3601101.f1.docx]

**Supplementary Table 1. Population doubling levels of the ASCs used for biological dressing production**

| **Passage** | **ASCs#1** | **ASCs#2** | **ASCs#3** |
| --- | --- | --- | --- |
| 1 | 4.95 | 4.04 | 4.82 |
| 2 | 3.62 | 3.90 | 4.23 |

**Supplementary Figure 1. All NONcNZO/LtJ10 and K14-H2B-GFP mice reached diabetic blood glucose level threshold.** A) Blood glucose levels of NONcNZO/LtJ10 mice at their arrival at the animal facility and after 13 weeks of high-fat diet. Data is shown according to the experimental group and each symbol represents an individual mouse. B) Blood glucose levels of K14-H2B-GFP mice before streptozotocin injection and 3 weeks after diabetes induction, n = 9-11 mice per group. Red dotted lines indicate diabetic blood glucose threshold set at 14.0 mM. Mean ± SEM, one-way ANOVA with Tukey post-hoc test, **** : p < 0.0001, * : p < 0.05.

**Supplementary Figure 2. ASC-derived biological dressings accelerate global wound closure notably in early phase in STZ-induced diabetic K14-H2B-GFP mice.** A) Macroscopic images of untreated (left column) and treated (right column) wounds at day (D) 0, D4, D8-9, D12-13, D17 and D20-22 after wound creation, scale bars: 0.5 cm. B) Kinetics of global wound closure percentage measured using macroscopic images, n = 20 wounds in the treated group and n = 6 - 16 wounds in the untreated group, mean ± SEM, unpaired t-test with Welch’s correction, * : p < 0.05, ** : p < 0.01, *** : p < 0.001. Two independent experiments were combined.


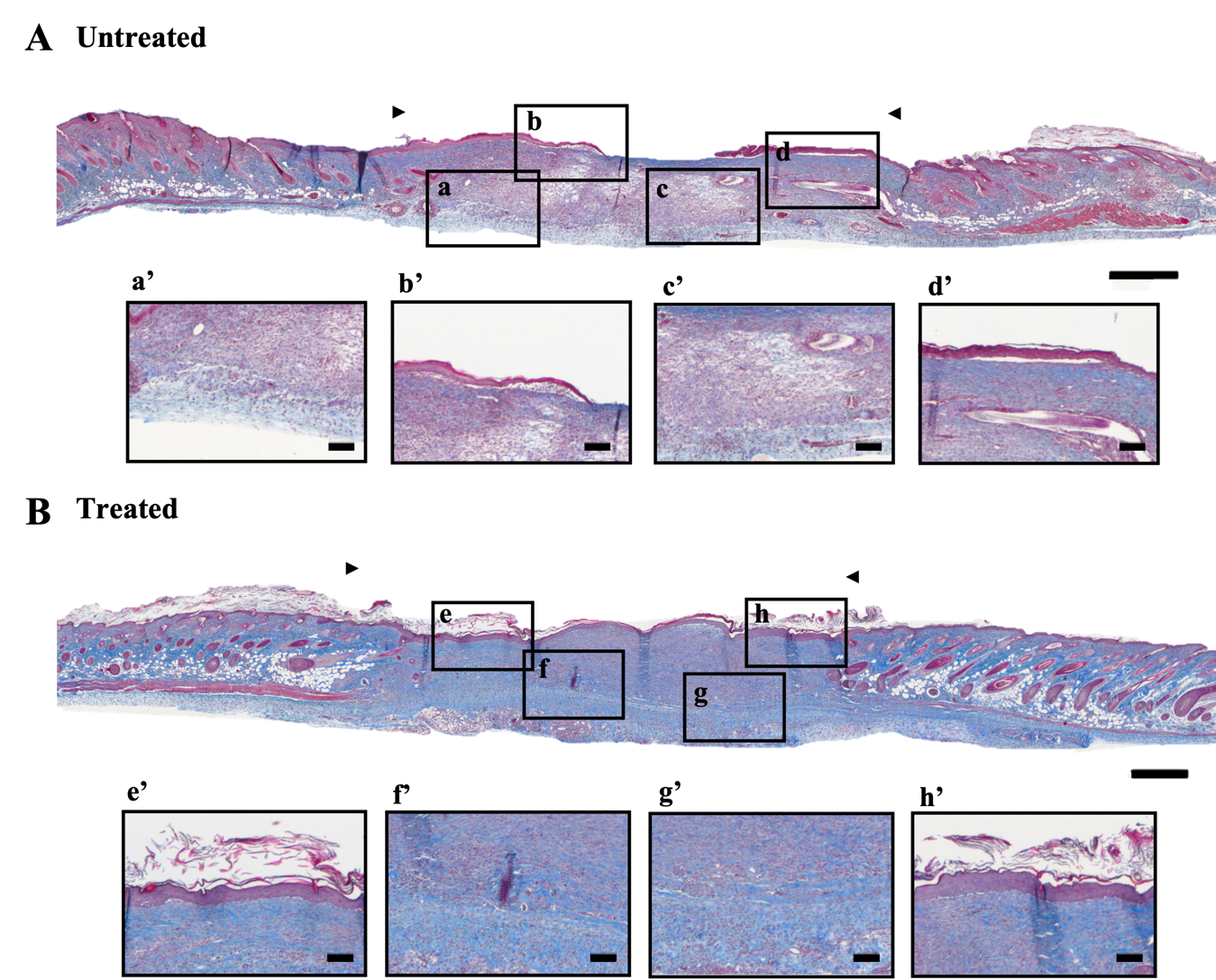


**Supplementary Figure 3. Wounds of STZ-induced diabetic K14-H2B-GFP mice treated with biological dressings display complete reepithelialization and homogenous granulation tissue.** Representative examples of Masson’s trichrome staining of K14-H2B-GFP mice wound/healed skin cross-sections for A) untreated wound 22 days after excisional wound surgery and for B) treated wound 21 days after surgery. Bars: 500 µm. The wound area is delimited by the arrowheads. (a’-f’) Magnifications of the neoepidermis (b, d, e, h) and granulation tissue (a, c, f, g) delimited by the black boxes (a-f) shown for each complete wound/healed skin section. Bars: 100 µm.


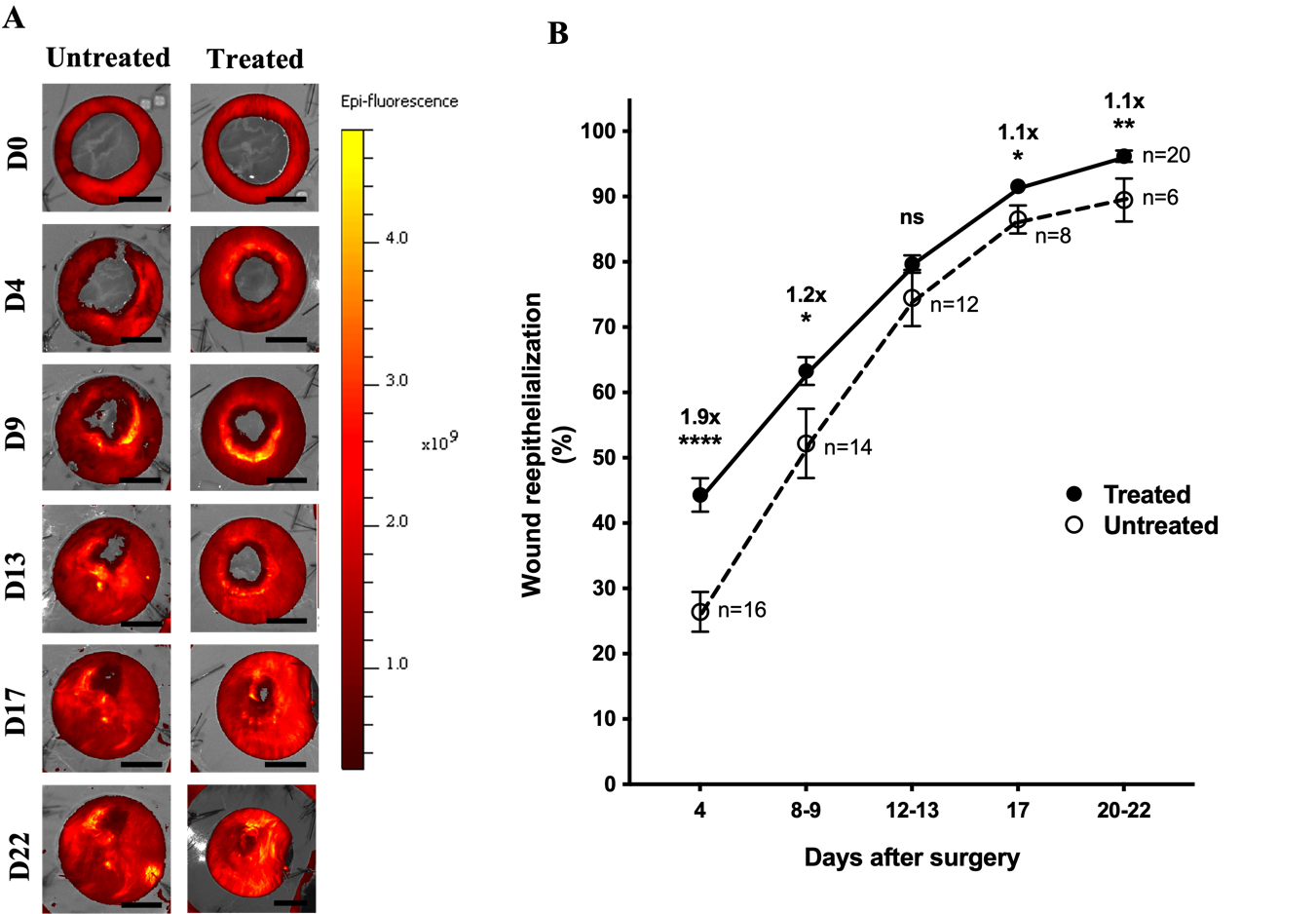


**Supplementary Figure 4. ASC-derived biological dressings accelerate early reepithelialization in STZ-induced diabetic K14-H2B-GFP mice.** A) *In vivo* fluorescence imaging of keratinocyte coverage at day (D) 0, D4, D9, D13, D17 and D22 after excisional wound surgery. Scale bars: 0.5 cm. B) Kinetics of wound reepithelialization measured from the fluorescence imaging data. Samples are n = 20 wounds in the treated group and n = 6-16 wounds in the untreated group as directly indicated on the graph, mean ± SEM, unpaired t-test with Welch’s correction, * : p < 0.05, ** : p < 0.01, **** : p < 0.0001. Two independent experiments were combined.
